# Epidemiological features of traumatic spinal cord injury in China: A systematic review and meta-analysis

**DOI:** 10.3389/fneur.2023.1131791

**Published:** 2023-03-20

**Authors:** Youpeng Hu, Lianxin Li, Binxue Hong, Yizhou Xie, Tong Li, Chaoqun Feng, Fei Yang, Yehui Wang, Jie Zhang, Yang Yu, Xiaohong Fan

**Affiliations:** ^1^The Affiliated Hospital of Chengdu University of Traditional Chinese Medicine, Chengdu, Sichuan, China; ^2^West China School of Medicine, West China Hospital, Sichuan University, Chengdu, Sichuan, China

**Keywords:** traumatic spinal cord injury, epidemiological factors, incidence, China, systematic review and meta-analysis

## Abstract

**Background:**

Traumatic spinal cord injury (TSCI) is a highly fatal and disabling event, and its incidence rate is increasing in China. Therefore, we collated the epidemiological factors of TSCI in different regions of China to update the earlier systematic review published in 2018.

**Method:**

We searched four English and three Chinese electronic databases from 1978 to October 1, 2022. From the included reports, information on sample characteristics, incidence, injury characteristics, prognostic factors, and economic burden was extracted. The selection of data was based on the PRISMA statement. The quality of the included studies was assessed by the Agency for Healthcare Research and Quality (AHRQ) tool. The results of the meta-analysis were presented in the form of pooled frequency and forest plots.

**Results:**

A total of 59 reports (60 studies) from 23 provinces were included, of which 41 were in the Chinese language. The random pooled incidence of TSCI in China was estimated to be 65.15 per million (95% CI: 47.20–83.10 per million), with a range of 6.7 to 569.7 per million. The pooled male-to-female ratio was 1.95:1. The pooled mean age of the cases at the time of injury was 45.4 years. Motor vehicle accidents (MVAs) and high falls were found to be the leading causes of TSCI. Incomplete quadriplegia and AISA/Frankel grade D were the most common types of TSCI. Cervical level injury was the most prevalent. The pooled in-hospital mortality and complication rates for TSCI in China were 3% (95% CI: 2–4%) and 35% (95% CI: 23–47%). Respiratory problems were the most common complication and the leading cause of death.

**Conclusion:**

Compared with previous studies, the epidemiological data on TSCI in China has changed significantly. A need to update the data over time is essential to implement appropriate preventive measures and formulate interventions according to the characteristics of the Chinese population.

## Introduction

### Rationale

Traumatic spinal cord injury (TSCI) is one of the most devastating and catastrophic injury types, with high mortality and disability rates, causing physical and emotional hardship to patients as well as imposing a significant burden on society and families (1–3). TSCI refers to injuries that damage neural structures in the spinal canal, such as the spinal cord, nerve roots, and cauda equine, due to traumatic factors (4). TSCI is usually accompanied by sensory, motor, reflex, defecation, and other dysfunctions (5). Disability resulting from TSCI may be permanent, and medical care may not be sufficient to abrogate it (6). Over the past 40 years, China has witnessed rapid urbanization and an increase in its aging population, which led to a noticeable increase in the TSCI (7–9).

### Objectives

This study aims to update the previous research (10) published in 2018 through systematic synthesis and meta-analysis. Toward this goal, we extracted the latest epidemiological data, categorized them based on the geographical divisions of China, and the differences between the North and South regions were evaluated. Additionally, by determining the risk factors for complications or premature death, this study could also improve public awareness of preventive measures and provide a framework for health resource allocation and policy formulation.

## Methods

### Design

This systematic review and meta-analysis of the literature were performed according to the PRISMA 2020 guidelines (11).

### Search strategy

We searched the original peer-reviewed studies from the earliest record in 1978 to October 1, 2022, in the following databases: PubMed, EMBASE, Web of Science, EBSCO, China National Knowledge Infrastructure (CNKI), Wan Fang Data, and the China Science and Technology Journal Database (VIP). The search strategy we employed is described in detail in Supplementary Table 1. We searched all the fields of the database records, combining the relevant epidemiological terms and TSCI-related terms. Since there were too many irrelevant documents in PubMed and EMBASE, we added the restrictive word “human.” Examples of search terms used for searching the Web of Science were: [(“spinal cord injury” or “Traumatic spinal cord injury”) and (epidemiology or incidence or etiology or prevalence) and China)]. We also checked the references of eligible studies, retrieved them, and identified any missing systematic reviews related to TSCI that were missing from the database search. In addition, we collected relevant summaries from the TSCI-related meeting minutes and checked the availability of the full text.

### Eligibility criteria

We used the CoCoPop model (condition, context, and population) as the inclusion structure instead of the traditional PICO approach (population, intervention, comparator, and outcome). Because it is more relevant to the issue of incidence and epidemiology (12). The inclusion and exclusion criteria are shown in Table 1.

**Table 1:** Summary of inclusion and exclusion criteria.

	**Inclusion**	**Exclusion**
Context	Any study published in any year, language or setting about TSCI in China	Reviews, animal studies, basic science studies, case reports or studies out of China
Population	All ages, occupations and genders	Specific ages (pediatric or geriatric), specific occupations (workers or drivers)
Condition	Sample characteristics (number of cases, mean age, male/female ratio, incidence), injury characteristics (etiology, severity of injury), prognostic factors (complications, in-hospital mortality, additional concurrent trauma), economic burden	Specific etiological focus (road traffic injuries, earthquake disaster), unrelated specific topics (depression, sleep disorder), specific injury level (cervical spine injury), non-traumatic spinal cord injury or singe traumatic spinal fracture

### Data selection and collection

Two authors (YH and LL) independently screened the title and abstract of each article according to the inclusion and exclusion criteria. The full text of the selected articles was evaluated, and data was extracted. The third author (TL) rechecked the accuracy and integrity of the extracted data before analysis. Any disagreements were settled by consensus or by the third author (TL).

### Data synthesis and analysis

We used the tabular summary method to synthesize the data from the systematic review (12). The “metan” function of STATA software version 16.0 was used to develop a moment-based random model for estimating the hazard ratio, pooled effect of the incidence, percentages of in-hospital mortality, and complications (13). Forest plots were drawn to visualize the heterogeneity and the results of the meta-analysis (14). I2 values obtained by Cochrane's Q test were used to evaluate the heterogeneity. The I2 values of 25, 50, and 75% correspond to low, medium, and high heterogeneity, respectively (15). We also performed a sensitivity analysis using case-by-case exclusion to assess the impact of individual studies on the overall meta-analysis estimates. Due to the high heterogeneity between the studies, we also conducted a subgroup analysis.

### Quality assessment

Since all included studies were cross-sectional, two independent authors (YH and LL) evaluated the quality of the included studies using the Agency for Healthcare Research and Quality (AHRQ) tool (Supplementary Table 2). The AHRQ tool assessed the risk of bias in five domains: selection bias, implementation bias, follow-up bias, measurement bias, and reporting bias. Further, it consists of 11 items, with a scoring system of 1 point for “yes” and 0 points for “no” or “unclear.” Based on the scores the studies were categorized as poor quality (0–3 points), medium quality (4–7 points), and good quality (8–11 points) (16). Disagreements, if any, were settled by consensus or by the third author (TL).

## Results

### Study selection and characteristics

**Figure 1:**
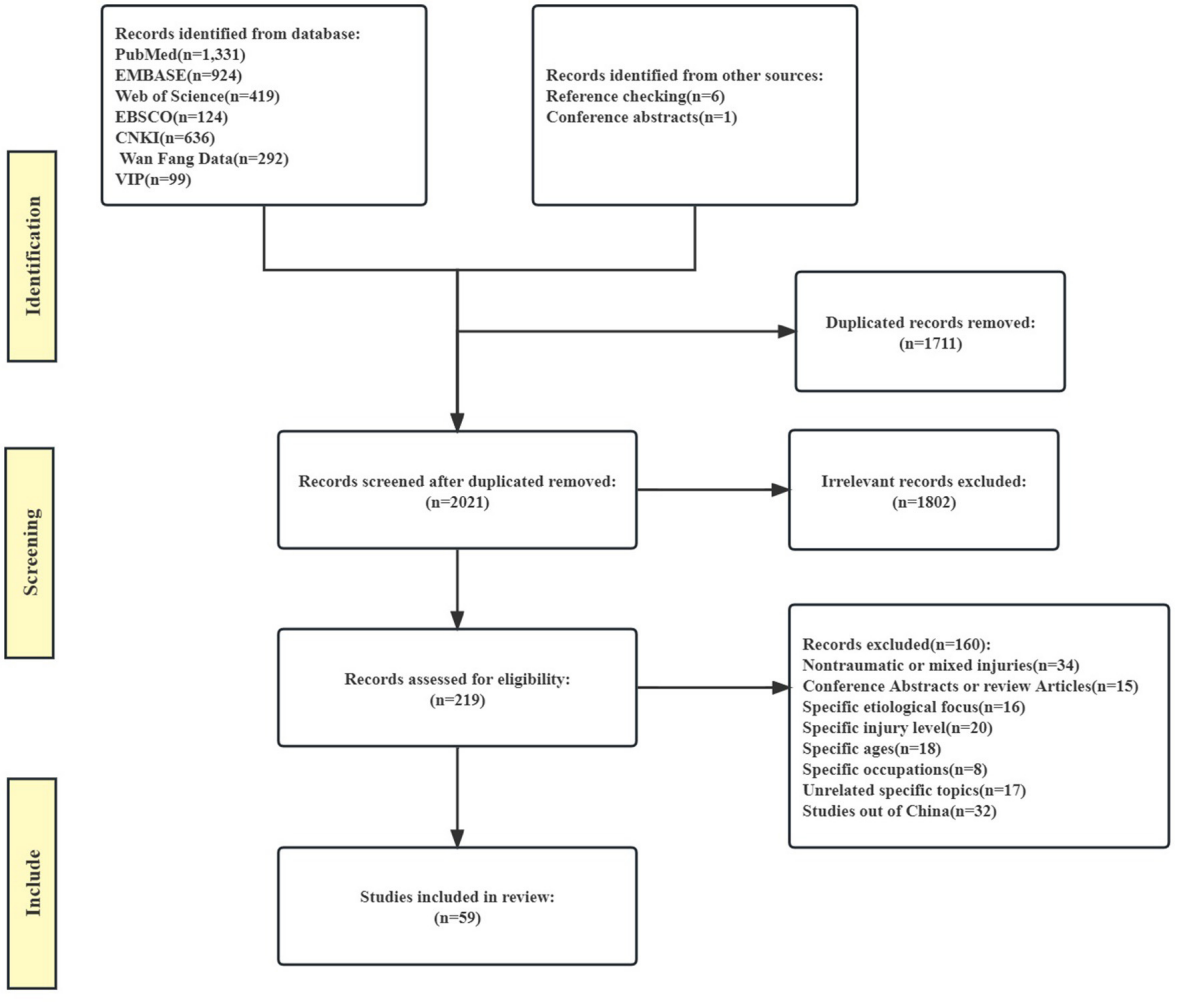
Flow diagram of each study stage.

We recognized a total of 3,825 records from the initial database search and pooled them into the EndNote X9 software. The flow diagram of each study stage according to PRISMA guidelines is illustrated in Figure 1. After reviewing the abstract and the full texts, 60 relevant studies were identified (^*^1–^*^59, ^*^52 contains two different studies, which were divided into ^*^52A and ^*^52B, see the Supplementary Appendix for the list of references).

### Methodological quality

We then performed a quality assessment of the included studies. The results of the quality assessment are summarized in Supplementary Table 3. Notably, almost all studies received a ”yes“ for questions on the sources and time of inclusion (items 1, 3). On the contrary, the questions about continuous sources (item 5) and the handling of follow-up and missing data (items 9–11) were answered ”yes“ less frequently. The total study score ranged from 2 to 7, with a median of 4. We assessed each study thoroughly for potential bias and did not exclude any studies.

### Sample characteristics

Table 2 displays the basic descriptive features of the 60 studies, including study authors, year of publication, region, incidence period, sample size, source population, source case, male to female ratio, and mean age. The publication period of the included studies spanned 44 years, with 40 studies published in the past 10 years (after 2012). Majority of studies were based on the review of hospital records, except for two studies (^*^53, ^*^54) which were based on national registers information. The maximum sample size was 54,484 from the Taiwan National Health Insurance (NHI) database (^*^53), while the smallest sample size was only 35 (^*^38, ^*^39). The mean age of TSCI cases at the time of injury was between 31.5 and 50.1 years, with a pooled mean age of 45.4 years. In all studies, the proportion of male patients was higher, with a highest male to female ratio of 15.3:1 (^*^13). And the pooled proportion of male to female ratio was 1.95:1 with a median of 4.0:1. We then extracted the top two occupations of TSCI patients from each study with largest number of TSCI patients, and found that the most vulnerable were workers, followed by farmers.

**Table 2:** Sample characteristics and etiology of TSCI in China.

	**References**	**Region**	**Incidence period**	**Source population**	**Case source**	**Total cases**	**Leading causes**	**Second causes**	**Gender ratio**	**Mean age**
North	Liu et al. (17)	Beijing	2017–2019	China rehabilitation research center	Hospitals records	252	MVAs	High fall	4.1:1	41.2
	Liu et al. (18)	Beijing	2013–2019	Beijing Boai Hospital	Hospitals records	2,448	Low fall	Assault	3.0:1	39.1 ± 16.7
	Cai et al. (19)	Tianjin	2013–2017	Three general hospitals in Tianjin city	Hospitals records	2,471	Low fall	High fall	2.9:1	49.2 ± 14.2
	Li et al. (20)	Inner Mongolia	2012–2019	The second affiliated hospital of Medical University	Hospitals records	956	High fall	MVAs	2.3:1	49.9 ± 20.7
	Wang et al. (21)	Beijing	2012–2015	PLA general hospital	Hospitals records	625	High fall	MVAs	4.5:1	38.2 ± 12.8
	Liu et al. (22)	Beijing	2011–2019	China rehabilitation research center	Hospitals records	590	High fall	MVAs	4.7:1	46.3 ± 15.5
	Yuan et al. (23)	Shanxi	2011–2014	Yuncheng central hospital	Hospitals records	58	MVAs	High fall	4.0:1	–
	Yang et al. (24)	Beijing	2009–2014	The first affiliated hospital of PLA general hospital	Hospitals records	1,027	MVAs	High fall	3.6:1	42.5 ± 12.4
	Zhou et al. (25)	Tianjin	2009–2014	General Hospital of Tianjin Medical University	Hospitals records	354	MVAs	Low fall	2.3:1	50.1 ± 15.5
	Xu et al. (26)	Beijing	2008–2011	Beijing Boai Hospital	Hospitals records	260	High fall		9.0:1	43.7
	Wang et al. (27)	Beijing	2005–2016	PLA General Hospital	Hospitals records	1,395	MVAs	High fall	4.1:1	32.1 ± 12.5
	Ning et al. (28)	Tianjin	2004–2008	Major general hospitals in Tianjin city	Hospitals records	869	Low fall	MVAs	5.6:1	46.0 ± 14.2
	Jiang et al. (29)	Beijing	2002–2011	The 322nd Hospital of the PLA	Hospitals records	423	Struck by object	High fall	15.3:1	40.0 ± 11.0
	Hua et al. (30)	Beijing	2001–2010	General Hospital of the Chinese Armed Police Force	Hospitals records	561	MVAs	High fall	4.1:1	34.7 ± 12.2
	Li et al. (31)	Tianjin	1999–2016	General Hospital of Tianjin Medical University	Hospitals records	735	MVAs	Low fall	2.9:1	49.7 ± 15.2
	Feng et al. (32)	Tianjin	1998–2009	Tianjin Medical University General Hospital	Hospitals records	239	Low fall	MVAs	4.6:1	45.4 ± 14.1
	Hao et al. (33)	Beijing	1992–2006	China Rehabilitation Research Center and Beijing Boai Hospital	Hospitals records	1,264	MVAs	High fall	4.0:1	34.9
	Diao et al. (34)	Beijing	1982–1986	A sample of spinal cord patients in Beijing hospitals	Hospitals records	310	High fall	Low fall	–	–
	Yu et al. (35)	Tianjin	2007	Major general hospitals in Tianjin city	Hospitals records	73	Low fall	MVAs	3.6:1	51.3 ± 14.6
	Wei et al. (36)	Beijing	2005	A sample of spinal cord patients in Beijing hospitals	Hospitals records	254	MVAs	High fall	2.3:1	41.0 ± 14.3
	Li et al. (37)	Beijing	2002	A sample of spinal cord patients in Beijing hospitals	Hospitals records	264	High fall	MVAs	3.1:1	41.7
Northeast	Liu et al. (38)	Liaoning	2013–2018	Seven hospitals in Shenyang and Xi'an	Hospitals records	2,416	High fall	Low fall	2.9:1	49.2 ± 14.4
	Ru et al. (39)	Liaoning	2010–2012	Eight general hospitals in Dalian	Hospitals records	1,155	MVAs	Low fall	2.4:1	50.1 ± 15.9
	Xu et al. (40)	Jilin	2010–2011	Jilin University Sino–Japanese Friendship Hospital	Hospitals records	1,274	Struck by object	High fall	2.3:1	43.6
	Chen et al. (41)	Heilongjiang	2009–2013	The Fourth Affiliated Hospital of Harbin Medical University	Hospitals records	232	MVAs	High fall	4.0:1	45.4 ± 14.4
Eastern	Niu et al. (42)	Jiangsu	2015–2019	The First Hospital of Soochow University	Hospitals records	422	MVAs	High fall	3.2:1	51.1 ± 14.2
	Tang et al. (43)	Shandong	2014–2019	Affiliated Hospital of Qingdao University	Hospitals records	332	–	–	3.7:1	49.2 ± 13.7
	Feng et al. (44)	Shandong	2013–2017	Liaocheng Peoples Hospital	Hospitals records	338	Low fall	MVAs	3.1:1	50.1 ± 14.1
	Wu et al. (45)	Jiangxi	2012–2018	The Affiliated Hospital of Nanchang University	Hospitals records	1,290	MVAs	Low fall	7.1:1	53.1 ± 16.2
	Niu et al. (46)	Jiangsu	2009–2014	Major general hospitals in Suzhou city	Hospitals records	859	High fall	MVAs	2.4:1	47.5 ± 15.5
	Wang et al. (47)	Anhui	2007–2010	Two general hospitals in Anhui Province	Hospitals records	761	High fall	MVAs	3.4:1	45.0
	Pan et al. (48)	Shanghai	2005–2007	Several hospitals in Pudong area	Hospitals records	200	High fall	MVAs	3.0:1	44.5
	Yang et al. (49)	Fujian	2004–2013	The 175th Hospital of the PLA	Hospitals records	1,089	High fall	MVAs	3.5:1	44.7
	Duan et al. (50)	Jiangxi	2003–2007	The First Affiliated Hospital of Nanchang University	Hospitals records	650	–	–	2.1:1	46.5
	Chen et al. (51)	Shandong	2002–2007	Affiliated Hospital of Qingdao University Medical College	Hospitals records	251	High fall	MVAs	3.5:1	40.4
	Hu et al. (52)	Shanghai	1983–1991	Shanghai Ruijin Hospital and Songjiang County People's Hospital	Hospitals records	153	High fall	MVAs	3.3:1	41.3
	Cheng et al. (53)	Shanghai	1977–2007	Shanghai Tongji University Hospital	Hospitals records	676	High fall	MVAs	1.7:1	42.2
	Sun et al. (54)	Shandong	2011	Affiliated Hospital of Qingdao University	Hospitals records	35	MVAs	High fall	10.7:1	50.1
	Feng et al. (55)	Jiangsu	1991	Six hospitals in Wuxi	Hospitals records	35	High fall	Struck by object	7.8:1	–
South	Zhang et al. (56)	Guangdong	2013–2018	Guangzhou Red Cross Hospital	Hospitals records	62	High fall	MVAs	2.7:1	36.0 ± 14.4
Central	Huang et al. (57)	Guangdong	2012–2016	Guangdong Work Injury Rehabilitation Hospital	Hospitals records	397	High fall	MVAs	4.0:1	40.1
	Yi et al. (58)	Hunan	2012–2014	Several general hospitals in Hunan	Hospitals records	1,274	Low fall	MVAs	2.3:1	43.6
	Deng et al. (59)	Hubei	2012–2014	Taihe Hospital Affiliated to Hubei Medical College	Hospitals records	424	High fall	MVAs	1.6:1	46.5
	Lv et al. (60)	Henan	2008–2017	Henan Provincial People's Hospital	Hospitals records	692	MVAs	High fall	2.6:1	46.3 ± 15.9
	Tang et al. (61)	Guangxi	2006–2010	Affiliated Hospital of Guangxi Medical University	Hospitals records	221	MVAs	High fall	6.4:1	38.3 ± 12.4
	Zhu et al. (62)	Hunan	2005–2009	Second Xiangya Hospital, Central South University	Hospitals records	163	MVAs	High fall	3.8:1	37.0 ± 10.9
	Yang et al. (63)	Guangdong	2003–2011	Several hospitals in Guangdong	Hospitals records	1,340	High fall	MVAs	3.5:1	41.6 ± 14.7
	Chen et al. (64)	Guangdong	1995–2010	Zhujiang Hospital of Southern Medical University	Hospitals records	286	High fall	MVAs	7.4:1	36.3 ± 10.1
Southwest	Ning et al. (65)	Chongqing	2009–2013	Chongqing Xinqiao Hospital	Hospitals records	554	High fall	MVAs	4.3:1	45.6 ± 13.8
	Mao et al. (66)	Sichuan	1996–2002	Huaxi Hospital of Sichuan University	Hospitals records	132	MVAs	High fall	2.5:1	31.5 ± 7.8
Northwest	Hao et al. (67)	Shaanxi	2011–2013	Xi'an Honghui Hospital	Hospitals records	2,565	Low fall	High fall	4.7:1	41.5 ± 11.2
	Zhang et al. (68)	Shaanxi	2018	Xi'an Honghui Hospital	Hospitals records	382	High fall	Low fall	3.0:1	50.0 ± 15.2
Taiwan	Yang et al. (69)	Taiwan	2000–2003	National Health Insurance (NHI) database	National register	54,484	–	–	1.0:1	–
	Wu et al. (70)	Taiwan	1998–2008	National Health Insurance (NHI) database	National register	41,586	MVAs	High fall	1.5:1	–
	Chen et al. (71)	Taiwan	1992–1996	Medical centers and general hospitals in Taiwan	Hospitals records	1 586	MVAs	High fall	3.0:1	46.1
	Lan et al. (72)	Taiwan	1986–1990	Four general hospitals in Taiwan	Hospitals records	99	MVAs	High fall	4.0:1	44.5
	Chen et al. (73)	Taiwan	1978–1981	Medical centers and general hospitals in Taiwan	Hospitals records	560	MVAs	High fall	4.9:1	36.2
Nationwide	Zhang et al. (68)	Whole	2018	National stratified whole group sampling	Hospitals records	4,404	High fall	Low fall	3.0:1	51.6 ± 15.3
	Hao et al. (74)	Whole	2018	National stratified whole group sampling	Hospitals records	4,134	MVAs	Low fall	3.0:1	50.8
	Jiang et al. (75)	Whole	2013	National stratified whole group sampling	Hospitals records	394	Low fall	MVAs	1.9:1	43.7 ± 17.1

These 60 studies include data from 23 provinces, representing about 1,129.4 million people (2020 census). The remaining 11 regions (Zhejiang, Yunnan, Guizhou, Xinjiang, Hainan, Gansu, Qinghai, Tibet, Ningxia, Hong Kong, and Macau) have not published any epidemiological studies related to TSCI. For the convenience of statistics and search, we categorized the studies into the following groups according to their geographical division: North (^*^1–^*^21), Northeast (^*^22–^*^25), East (^*^26–^*^39), South Central (^*^40–^*^48), Southwest (^*^49–^*^50), Northwest (^*^51–^*^52A), Taiwan (^*^53–^*^57), and Nationwide (^*^52B, ^*^58–^*^59).

### Injury characteristics

Following these preliminary analyses, we looked at the characteristics of the TSCI cases. We found that the most frequent causes of TSCI were motor vehicle accidents (MVAs), which accounted for 40.7% of all the cases, followed by high falls (39.8%). The detailed causes of TSCI are summarized in Table 2. The level of injury was reported in forty-four studies, which showed the majority of injuries occurred at the cervical level, followed by the lumbosacral level (Table 3). Of note, 41 studies used the AISA/Frankel grade in describing injury severity, while 21 studies measured the extent of the injury (complete or incomplete) and the neurological level of the injury (tetraplegia or paraplegia). Eleven studies did not report the severity of injuries. According to 24 studies, AISA D was the most common grade with a highest proportion of 68.4% (^*^23), followed by grade A, as reported by 13 studies. Further, incomplete injuries were more prevalent than complete injuries, with incomplete quadriplegia being the most common.

**Table 3:** Level and severity of TSCI in China.

**References**	**C (%)**	**T (%)**	**L (%)**	**AISA A (%)**	**AISA B (%)**	**AISA C (%)**	**AISA D (%)**	**AISA E (%)**	**IQ (%)**	**IP (%)**	**CP (%)**	**CQ (%)**
Liu et al. (17)	47.2	43.3	9.5	48.0	15.1	14.3	22.6	–	–	–	–	–
Liu et al. (18)	–	–	–	–	–	–	–	–	–	–	–	–
Cai et al. (19)	63.7	21.1	15.1	37.9	9.6	13.4	39.1	–	37.2	18.9	25.6	18.3
Li et al. (20)	52.9	31.5	15.6	43.5	12.5	23.0	21.0	–	–	–	–	–
Wang et al. (21)	32.8	51.0	16.2	52.5	14.7	17.0	15.0	0.8	17.9	29.0	38.1	15.0
Liu et al. (22)	54.9	32.7	12.4	33.1	13.6	24.5	28.8	–	–	–	–	–
Yuan et al. (23)	55.0	17.0	28.0	66.0	14.0	10.0	10.0	–	–	–	–	–
Yang et al. (24)	–	–	–	34.6	7.9	17.7	16.2	3.3	–	–	–	–
Zhou et al. (25)	59.3	22.0	18.7	20.4	7.6	23.2	48.8	–	–	–	–	–
Xu et al. (26)	14.6	53.8	31.6	80.8	16.2	3.0	0.0	–	19.2#	–	80.8#	–
Wang et al. (27)	–	–	–	52.2	11.8	15.0	21.0	–	–	–	–	–
Ning et al. (28)	71.5	–	–	25.2	18.2	14.7	41.9	–	–	–	–	–
Jiang et al. (29)	–	–	–	45.4	3.1	17.7	30.0	3.8	–	–	–	–
Hua et al. (30)	–	–	–	–	–	–	–	–	–	–	–	–
Li et al. (31)	–	–	–	–	–	–	–	–	52.5	23.8	8.3	15.4
Feng et al. (32)	82.0	–	–	32.6	12.1	16.3	38.9	0.0	54.4	22.6	10.1	7.9
Hao et al. (33)	31.5	21.4	28.1	–	–	–	–	–	43.3#	–	56.7#	–
Diao et al. (34)	–	–	–	–	–	–	–	–	–	–	–	–
Yu et al. (35)	83.6	9.6	6.8	26.4	11.1	18.1	43.1	1.4	–	–	–	–
Wei et al. (36)	31.9	21.3	8.7	–	–	–	–	–	46.9#	–	35.8#	–
Li et al. (37)	4.9	28.0	66.7	–	–	–	–	–	–	–	–	–
Liu et al. (38)	55.1	29.9	14.9	29.8	5.0	10.9	54.3	–	39.1	–	–	–
Ru et al. (39)	57.6	14.7	27.7	19.6	2.4	9.6	68.4	–	52.7	31.5	8.3	6.3
Xu et al. (40)	32.5	19.5	52.8	–	–	–	–	–	–	–	–	–
Chen et al. (41)	76.3	10.3	13.4	14.2	15.1	32.8	37.9	–	63.4	18.5	5.2	12.9
Niu et al. (42)	69.4	12.8	17.8	–	–	28.7	52.1	–	–	–	–	–
Tang et al. (43)	56.4	35.2	35.2	25.8	19.8	24.6	29.9	–	–	–	–	–
Feng et al. (44)	77.2	–	–	29.3	5.3	16.3	48.5	0.6	–	–	–	–
Wu et al. (45)	–	–	–	13.4	19.2	27.2	40.2	–	–	–	–	–
Niu et al. (46)	43.2	11.5	33.9	19.4	5.2	31.5	36.9	4.2	–	–	–	–
Wang et al. (47)	46.3	20.4	33.3	25.6	11.8	27.3	35.2	–	–	–	–	–
Pan et al. (48)	29.0	35.0	36.0	17.5	20.0	38.5	24.0	–	–	–	–	–
Yang et al. (49)	63.0	19.5	17.5	29.6	7.0	18.9	27.0	17.5	61.3	6.2	15.2	17.3
Duan et al. (50)	41.7	7.1	51.2	–	–	–	–	–	51.7#	–	48.3#	–
Chen et al. (51)	29.1	4.8	36.7	27.1	4.0	17.5	51.4	–	–	–	–	–
Hu et al. (52)	–	–	–	–	–	–	–	–	–	21.6	18.3	–
Cheng et al. (53)	17.4	27.0	55.6	17.6	4.9	12.0	38.7	26.8	–	–	–	–
Sun et al. (54)	–	–	–	34.3	20.0	20.0	25.7	–	65.7#	–	34.3#	–
Feng et al. (55)	31.4	34.3	34.3	17.1	42.9	17.1	22.9	–	–	–	–	–
Zhang et al. (56)	53.2	30.7	16.1	27.4	22.6	12.9	21.0	16.1	–	–	–	–
Huang et al. (57)	39.8	39.6	20.6	–	–	–	–	–	85.9#	–	14.1#	–
Yi et al. (58)	51.7	3.8	40.7	11.5	4.0	31.9	34.9	0.4	42.5	9.2	15.2	12.3
Deng et al. (59)	20.2	31.5	48.3	9.4	1.7	4.5	27.8	56.6	–	–	–	–
Lv et al. (60)	70.1	11.7	17.8	24.1	19.3	15.8	40.8	–	54.4	19.3	9.2	17.1
Tang et al. (61)	56.6	21.2	22.2	23.5	18.6	26.7	31.2	–	–	–	–	–
Zhu et al. (62)	25.2	36.2	38.7	25.2	49.1	21.5	4.3	–	–	–	–	–
Yang et al. (63)	56.7	20.5	22.8	–	–	–	–	–	73.6#	–	26.4#	–
Chen et al. (64)	28.7	46.9	24.4	–	–	–	–	–	38.8#	–	61.2#	–
Ning et al. (65)	54.0	30.3	15.7	39.4	8.7	21.0	30.8	–	–	–	–	–
Mao et al. (66)	–	–	–	42.0	13.0	29.0	16.0	–	–	–	–	–
Hao et al. (67)	51.8	14.1	34.1	27.8	16.2	11.5	36.7	7.8	–	–	–	–
Zhang et al. (68)	–	–	–	15.7	7.9	19.7	48.3	8.4	–	–	–	–
Yang et al. (69)	–	–	–	–	–	–	–	–	–	–	–	–
Wu et al. (70)	–	–	–	–	–	–	–	–	–	–	–	–
Chen et al. (71)	49.9	13.3	34.6	–	–	–	–	–	–	–	–	–
Lan et al. (72)	–	–	–	–	–	–	–	–	–	–	–	–
Chen et al. (73)	46.8	–		–	–	–	–	–	–	–	–	–
Zhang et al. (68)	63.4	24.2	12.4	19.5	10.4	27.9	42.2	–	80.4#	–	19.6#	–
Hao et al. (74)	64.5	12.1	23.4	–	–	28.0	43.66	–	55.2	26.6	–	–
Jiang et al. (75)	–	–	–	–	–	–	–	–	–	–	–	–

C, Cervical; T, Thoracic; L, Lumbosacral; IQ, Incomplete Quadriplegia; IP, Incomplete Paraplegia; CQ, Complete Quadriplegia; CP, Complete Paraplegia; # means that only complete or incomplete injuries were reported.

AISA A-E; AISA/Frankel grade A-E.

### Incidence

**Figure 2:**
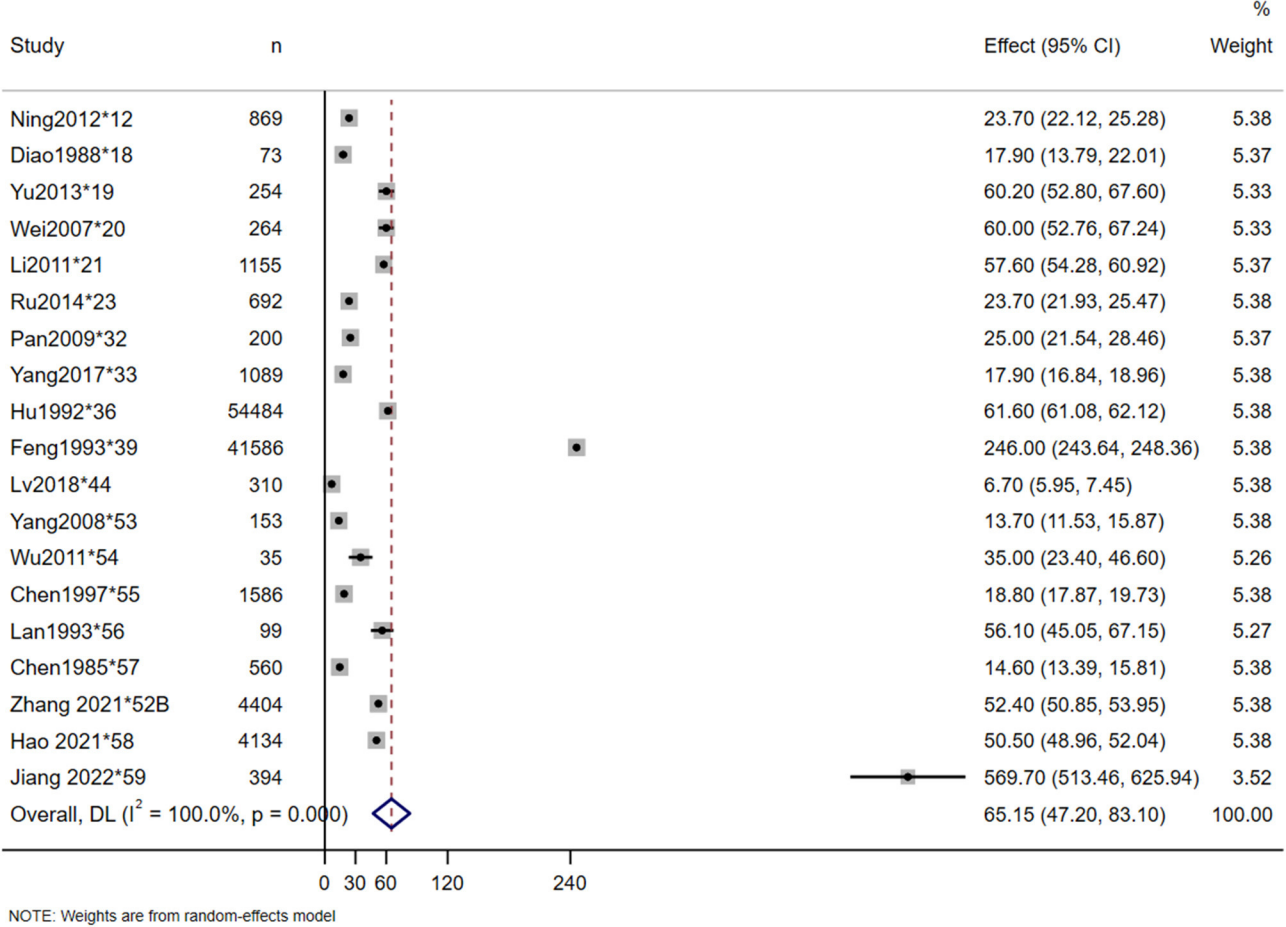
Incidence of TSCI meta-analysis in nationwide.

**Figure 3:**
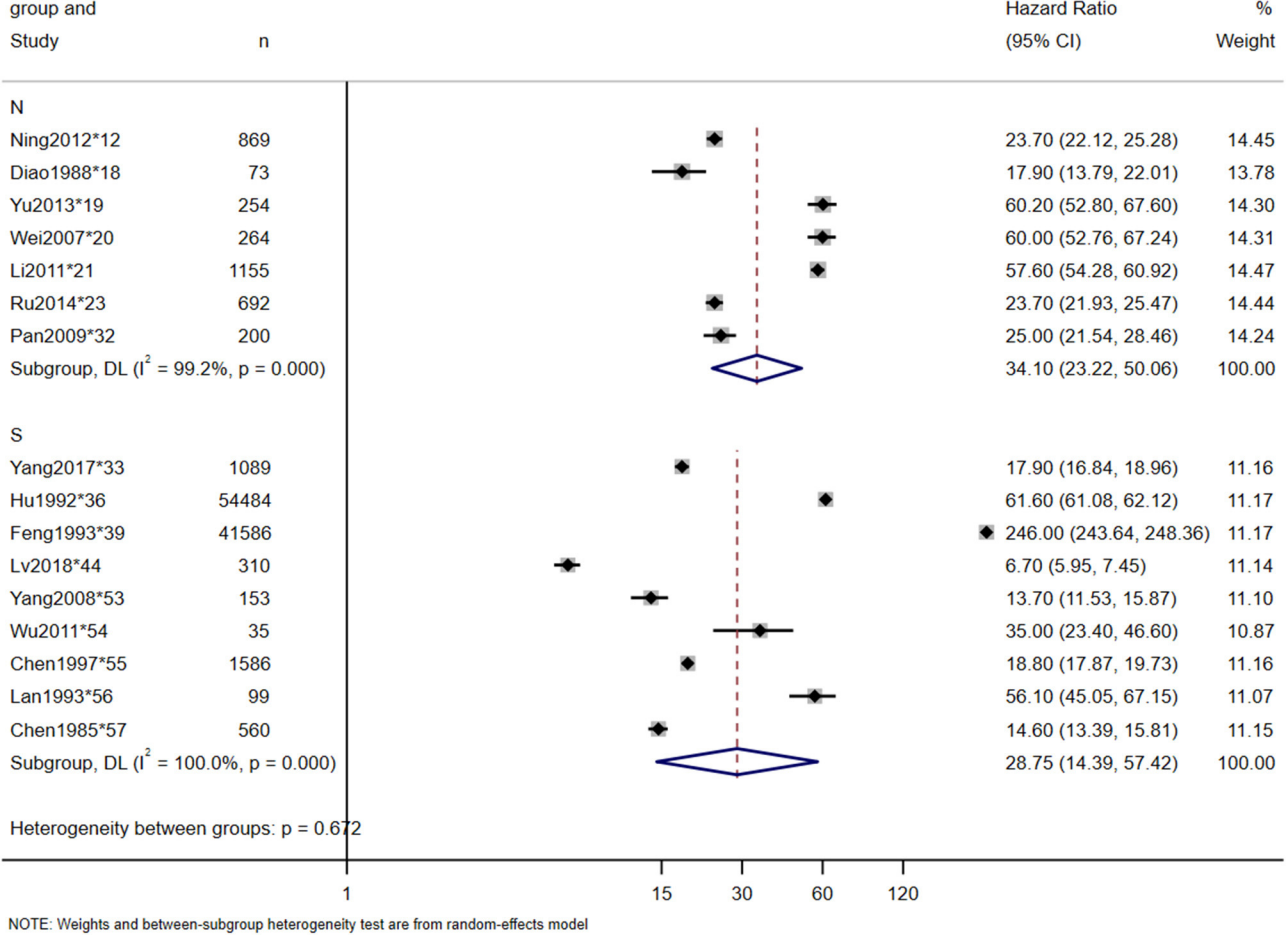
Incidence of TSCI meta-analysis in North-South subgroup.

The incidence rates in this study were calculated by dividing the number of new-onset TSCI cases in a given area during a given period of time by the total at-risk population during the same period. A total of 19 studies reported the incidence of TSCI, which ranged from 6.7 per million in 1988 (^*^18) to 569.7 per million in 2022 (^*^59). The estimated incidence rate of TSCI in China was 65.15 per million (95% CI: 47.20–83.10 per million, heterogeneity test: I2 = 100%, *p*-value = 0) (Figure 2). Owing to the high heterogeneity between the studies, we also conducted a North-South subgroup analysis to explore the causes. In the subgroup analysis, a total of 7 and 9 studies were included in the North and South subgroups, respectively, and 3 studies that were conducted nationwide were excluded. The subgroup analyses showed that the incidence of TSCI in the North was 34.10 per million (95% CI: 23.22–50.06 per million, heterogeneity test: I2 = 99.2%, *p*-value = 0), and in the South was 28.75 per million (95% CI: 14.39–57.42 per million, heterogeneity test: I2 = 100%, *p*-value = 0) (Figure 3). The hazard ratio's pooled effect was used to estimate the incidence rate. Heterogeneity test between groups: *p*-value = 0.672. We also performed a sensitivity analysis using case-by-case exclusion, and no study was excluded (Supplementary Figure 1).

### Prognostic factors

**Figure 4:**
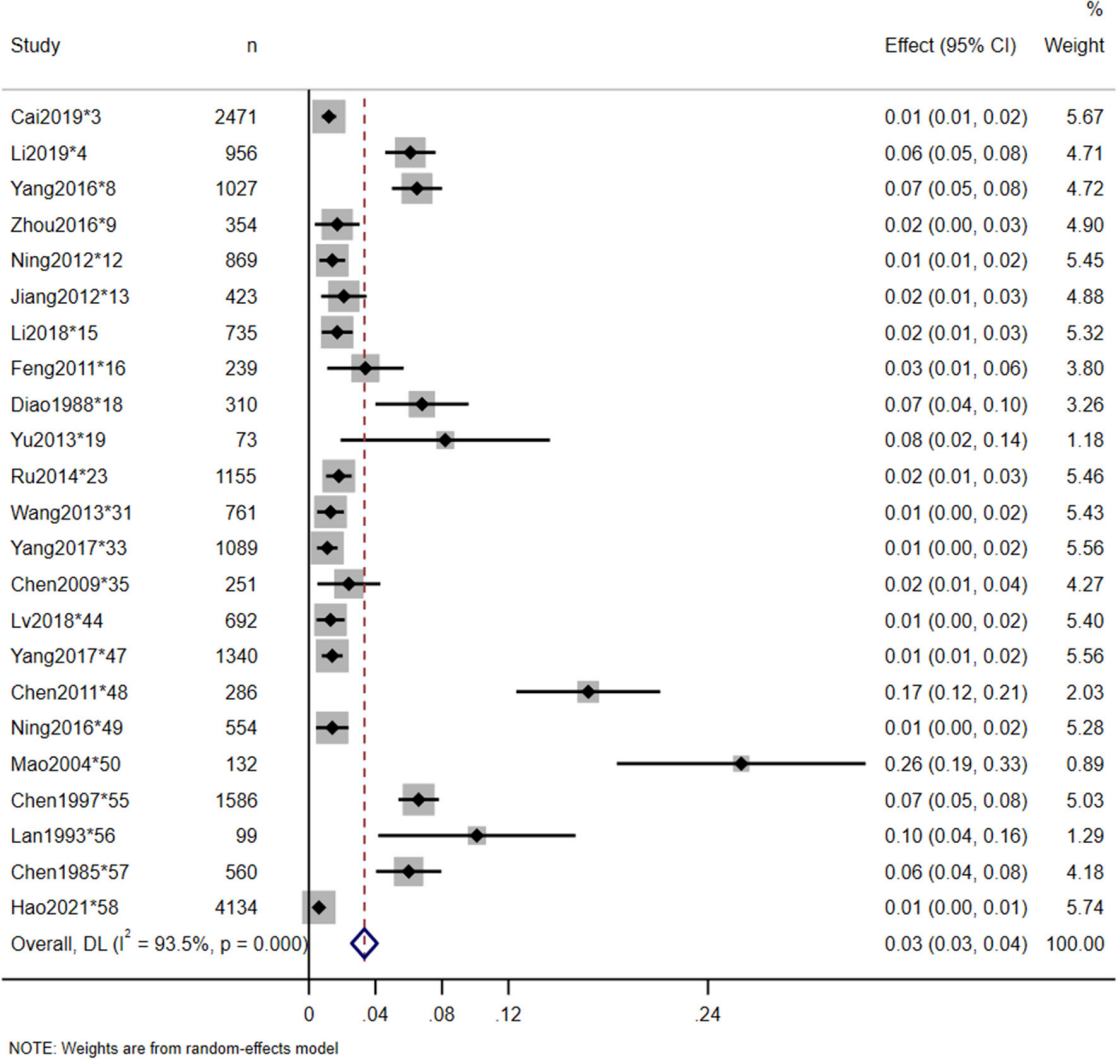
In-hospital mortality of TSCI meta-analysis in nationwide.

**Figure 5:**
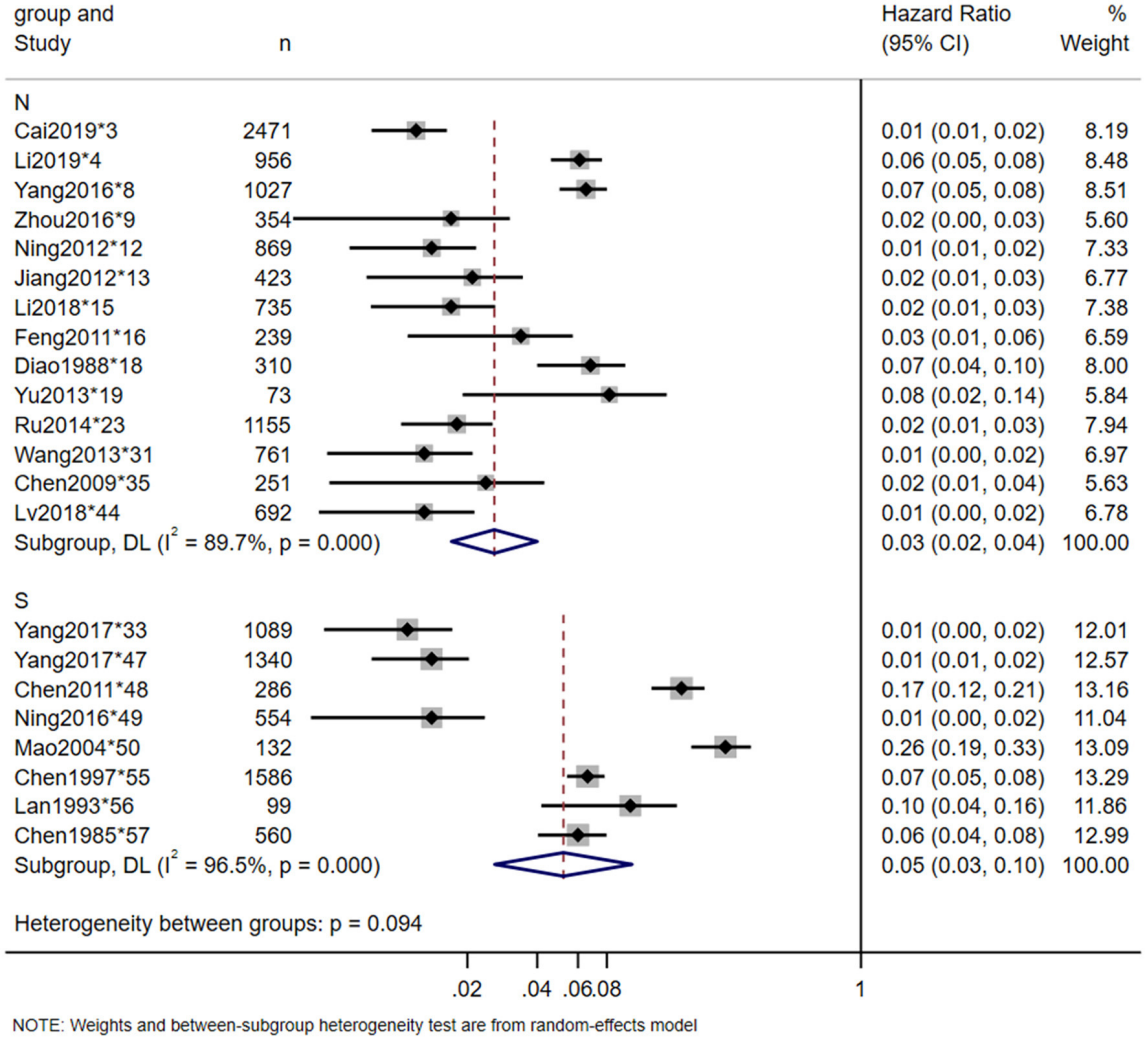
In-hospital mortality of TSCI meta-analysis North-South subgroup.

**Figure 6:**
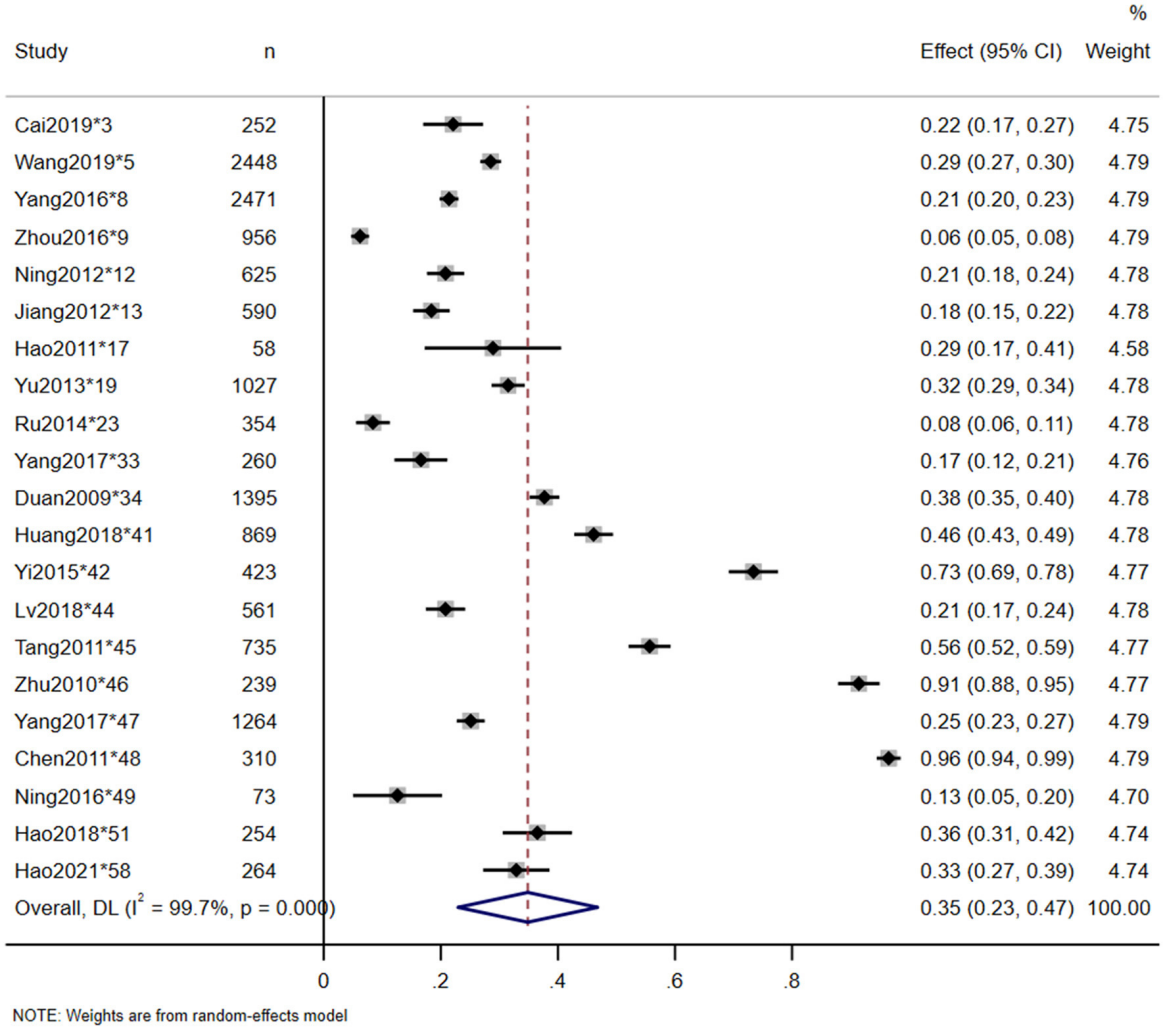
Complication rate of TSCI meta-analysis in nationwide.

**Figure 7:**
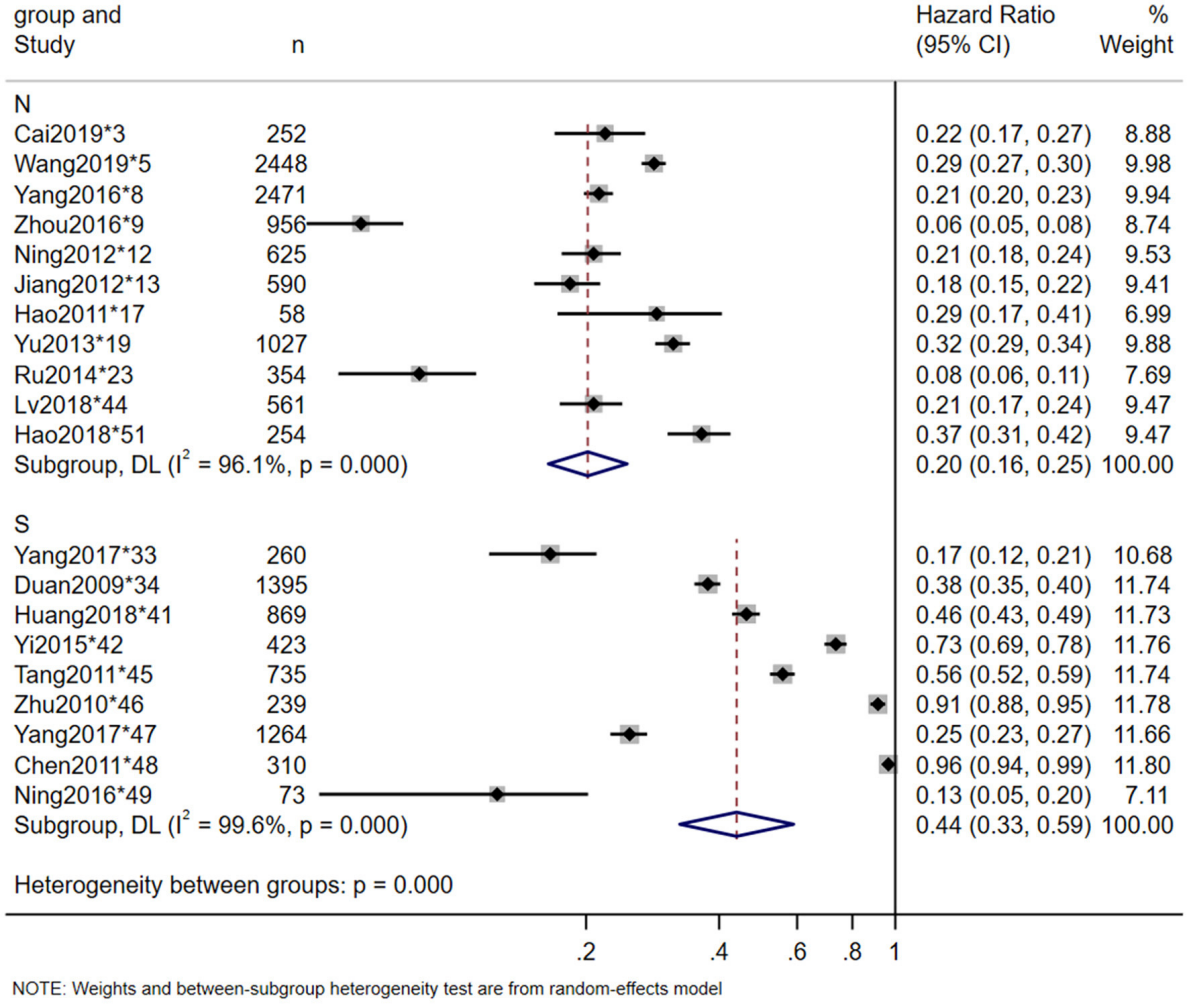
Complication rate of TSCI meta-analysis in North-South subgroup.

Twenty-three studies reported the in-hospital mortality of TSCI, and 20 studies reported the major causes of death. The pooled estimate for the in-hospital mortality of TSCI in China was 3% (95% CI: 3–4%, heterogeneity test: I2 = 93.5%, *p*-value = 0) (Figure 4). In the subgroup analysis, the estimation of TSCI in-hospital mortality in the North was 3% (95% CI: 2–4%, heterogeneity test: I2 = 89.7%, *p*-value = 0), and in the South was 5% (95% CI: 3–10%, heterogeneity test: I2 = 96.5%, *p*-value = 0). Heterogeneity test between groups: *p*-value = 0.094 (Figure 5). The most frequent cause of death from TSCI was respiratory failure, as reported by 15 studies. A total of 11 studies mentioned the incidence of additional concurrent trauma, ranging from 51.0% (^*^15) to 94.6% (^*^51), the pooled estimate for the proportion of additional concurrent trauma in China was 71% (95% CI: 60–81%, heterogeneity test: I2 = 99.6%, *p*-value = 0) (Supplementary Figure 2). The main additional concurrent trauma was spinal fracture. Thirty studies mentioned complications from TSCI, among which respiratory diseases and urinary diseases were the most common. Further, 21 studies reported the complication rate of TSCI, ranging from 6.2% (^*^9) to 96.5% (^*^48). Our analysis shows that the pooled estimate for the proportion of in-hospital mortality in China was 35% (95% CI: 23–47%, heterogeneity test: I2 = 99.7%, *p*-value = 0) (Figure 6). Our subgroup analysis in Figure 7, shows that the in-hospital mortality in the North was 20% (95% CI: 16–25%, heterogeneity test: I2 = 96.1%, *p*-value = 0) and in the South was 44% (95% CI: 33–59%, heterogeneity test: I2 = 99.6%, *p*-value = 0). Heterogeneity test between groups: *p*-value = 0. No study was excluded due to sensitivity analysis (Supplementary Figures 3, 4).

### Economic burden

Twelve studies mentioned the economic burden of TSCI, 10 of which mentioned the average hospitalization costs, ranging from 4.1 thousand RMB in 1988 (^*^18) to 252.3 thousand RMB in 2020 (^*^1). The pooled estimate for the economic burden of TSCI was 48.5 thousand RMB.

## Discussion

In this study, we conducted the first meta-analysis of the epidemiological data on TSCI in China and updated the previous study published in 2018 (10). We searched 7 databases and identified 59 papers (60 studies), representing 23 provinces and about 1,129.4 million people. Three studies reported epidemiological data through stratified sampling nationwide. While 11 provinces have no reports of TSCI epidemiology, 15 provinces (65.2%) have only 1–2 studies. Among all the reports, 19 studies, all from Beijing and Tianjin, accounted for 31.7% of the total. The imbalance in the number of publications in different provinces has significantly affected the analysis of the national epidemiological data. Taking this disparity into account, along with the difference in economic levels in different regions of China, we conducted a North-South region-wise subgroup analysis to explore the sources of heterogeneity in the meta-analysis. Based on the region, we included thirty-three studies in the northern group and 24 in the southern group. The demarcation between north and south China is often considered to be the Qinling mountain range and the Huaihe river. The economic levels of the North and South had an obvious disparity revealed by the gross domestic product (GDP) of the South, which accounted for about 62% of the national total in 2018. Moreover, the industrial landscape is quite different between these regions. The northern region was biased toward heavy industry, while the southern region vigorously developed the economy through software and internet services industry. However, the net inflow of population in large cities in the southern region is on the rise, presenting a pattern of population flow from north to south, accompanied by a significantly faster urbanization rate compared to the northern region.

Our data points out that the annual incidence of TSCI ranged from 6.7 to 569.7 per million in China, and the random pooled incidence was estimated to be 65.15 per million. The annual incidence of TSCI in China reported in 2018 ranged from 13 to 60 per million (10), while the incidence of TSCI in Asia reported in 2012 ranged from 12.06 to 61.6 per million (76). Furthermore, the global TSCI incidence in 2011 was estimated to be 23 per million (77). Compared with other studies, the incidence of TSCI in China is higher than that in Asia and the rest of the world and still exhibiting an increasing trend. In the subgroup analysis, the estimated incidence of 28.75 per million in the south is lower than that of 34.10 per million in the north and much lower than the national average. This reduction is explained by the exclusion of three nationwide studies, especially the 569.7 per million reported in 2013(^*^52B). Of note, the industrial structure in the north is biased toward heavy industry, which exposes workers to high-risk environments and could explain the increased incidence of TSCI in the north compared to the south. Coal miners, for example, were the most common occupation for those with TSCI in Tianjin (^*^13). Comparing the incidence rate of TSCI at different time points in the same province, for example, the incidence rate of Beijing was 6.7 per million (^*^ 18) in 1988 to 60.2 per million (^*^ 20) in 2007, we can find an increasing TSCI incidence over time.

We found that the mean age at the time of injury reported worldwide was 33 years, down from 45.4 years in this study. We also noticed a correlation between increasing age and TSCI when comparing studies conducted in the same province at different time points. This may be related to China's transition toward an aging society, where a higher proportion of people over the age of 35 are engaging in high-risk occupations (78). Moreover, we found that there was a significant gender difference in TSCI incidence. The proportion of male TSCI patients was higher in almost all studies, with the highest male-to-female ratio of 15.3:1 (^*^13) and the pooled estimate for the male-to-female ratio was 1.95:1. This could be due to male workers engaging in high-risk work, such as truck driving and high-altitude construction work, more than their female counterparts. In addition, male drivers are more likely to engage in risky behaviors. For example, Chen et al. reported violence and alcoholism as potential causes for TSCI (^*^57).

In our analysis, motor vehicle accidents and high falls were the most common etiologies of TSCI, which was consistent with the 2018 study (10). This is related to the increase in the usage of private cars, urbanization, and the rapid economic growth after a change in China's economic policies. In the severity assessment of TSCI, the AISA/Frankel grade is the most commonly used method for classification. Most patients were classified as grade A in Asia (76) and in previous study (10). On the contrary, we found that grade D was the most prevalent, followed by grade A. This is also consistent with our finding that the most common neurological injury associated with TSCI was incomplete quadriplegia. This could be because of the increase in the aging population in China and their associated lifestyle of staying alone resulting in an increased chance of low falls. In this study, low fall was found to be the third most common cause of TSCI.

The report on in-hospital mortality and complications can help us understand the risks of TSCI, guide treatment to reduce and avoid complications, and ultimately achieve the goal of reducing mortality. The pooled in-hospital mortality rate in this study was estimated at 3%, and the main cause of death was respiratory failure. Some studies mentioned the death of respiratory failure due to cervical spinal cord compression in the acute stage, and the death of pulmonary infection after tracheotomy due to respiratory system related complications in the subacute stage. But this may be an underestimate, as up to 46% of injury deaths on death certificates between 2006 and 2016 did not have an S or T code (N code) for the nature of the injury (79). Besides, the number of TSCI patients who may have died at the scene or en route to the hospital is undetermined and the tradition in some regions of China is to take seriously ill patients home to spend the last time with their families or give up treatment due to financial burden. The high disability rate of TSCI is not only reflected in nervous system damage, but also in the increased complications. The complication rate of TSCI in China was estimated at 35%, and the major secondary complications were respiratory and urinary diseases. However, after comparing the results with the developing countries (80), the results showed that pressure ulcers and urinary tract infections were the most common. In the subgroup analysis, the in-hospital mortality and complication rates in the south were higher than those in the north. Since the main complications and causes of death were respiratory related diseases, the difference between the south and north may be related to the difference in climate or medical factors.

Our study has several limitations, as follows: (1) Most of the reports we included were retrospective studies of hospital records in individual provinces, with very limited community-based studies, and hence may suffer from publication bias. (2) The diagnostic criteria of these studies were inconsistent and lacked objective indicators such as MRI rates. Most studies only used AISA/Frankel grade to evaluate, so it was difficult to conduct a meta-analysis in such instances. (3) This study focused on TSCI survivors, which excluded those who died before reaching the hospital or who returned home due to tradition or economic burden, and there were few studies describing the additional concurrent trauma at admission, which may have an impact on the outcome of the study. (4) It is unclear whether the hospitals that conducted the study are representative of the region or whether there are other hospitals in the region that also treat TSCI, leaving a possibility that the epidemiological data may be inaccurate. (5) There were few large-scale national epidemiological surveys on TSCI, and these studies were mainly concentrated in the provinces with better resources. Due to the aforementioned limitations, accurate epidemiological data on TSCI are difficult to obtain in China.

## Conclusion

Traumatic spinal cord injury can usually be reduced by early prevention, and the government should issue appropriate policies based on epidemiological survey data and the different regions in the north and south. The increasing incidence of TSCI in China suggests that an urgent emphasis on prevention of the occurrence of TSCI in high-risk occupations and prevention of treatment complications is required. It is proposed that the standardization of TSCI epidemiological reports should be established in the future. Future research that are prospective, nationwide, and multicenter are required for establishing the epidemiology and TSCI. Finally, we hope that this review can provide guidance for traumatic spinal cord injury prevention, treatment, and rehabilitation in China.

## Author contributions

YH and LL conceived the idea and performed data collection and extraction. TL and CF contributed to data inspection and synthesis. YH, YX, FY, and BH analyzed the data and wrote the manuscript. JZ and YW completed the critical review of manuscript. YY and XF supervised the project. All authors discussed the results and and approved the final version for publication.
