# Supplementary material for: Epidemiological features of traumatic spinal cord injury in China: A systematic review and meta-analysis
Source: Front Neurol. 2023 Mar 20;14:1131791. doi: 10.3389/fneur.2023.1131791 (PMC10069652; doi:10.3389/fneur.2023.1131791)
Supplement: Supplementary file 1 [file Data_Sheet_1.docx]

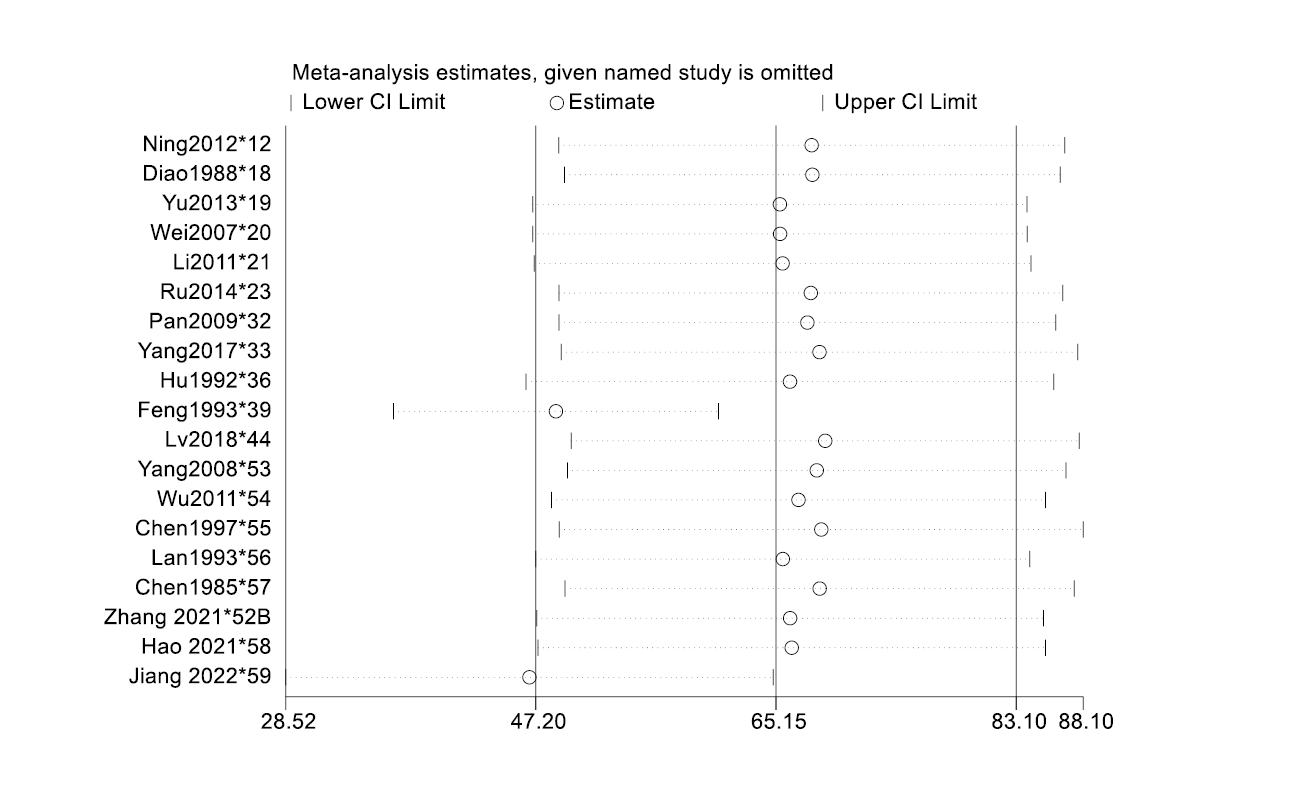


Supplementary Figure 1 Sensitivity analysis for incidence of TSCI in China


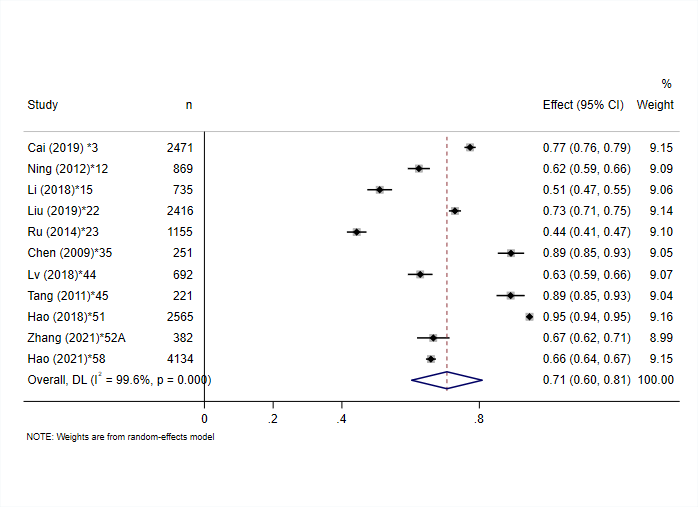


Supplementary Figure 2 Additional concurrent trauma rate of TSCI meta-analysis in China


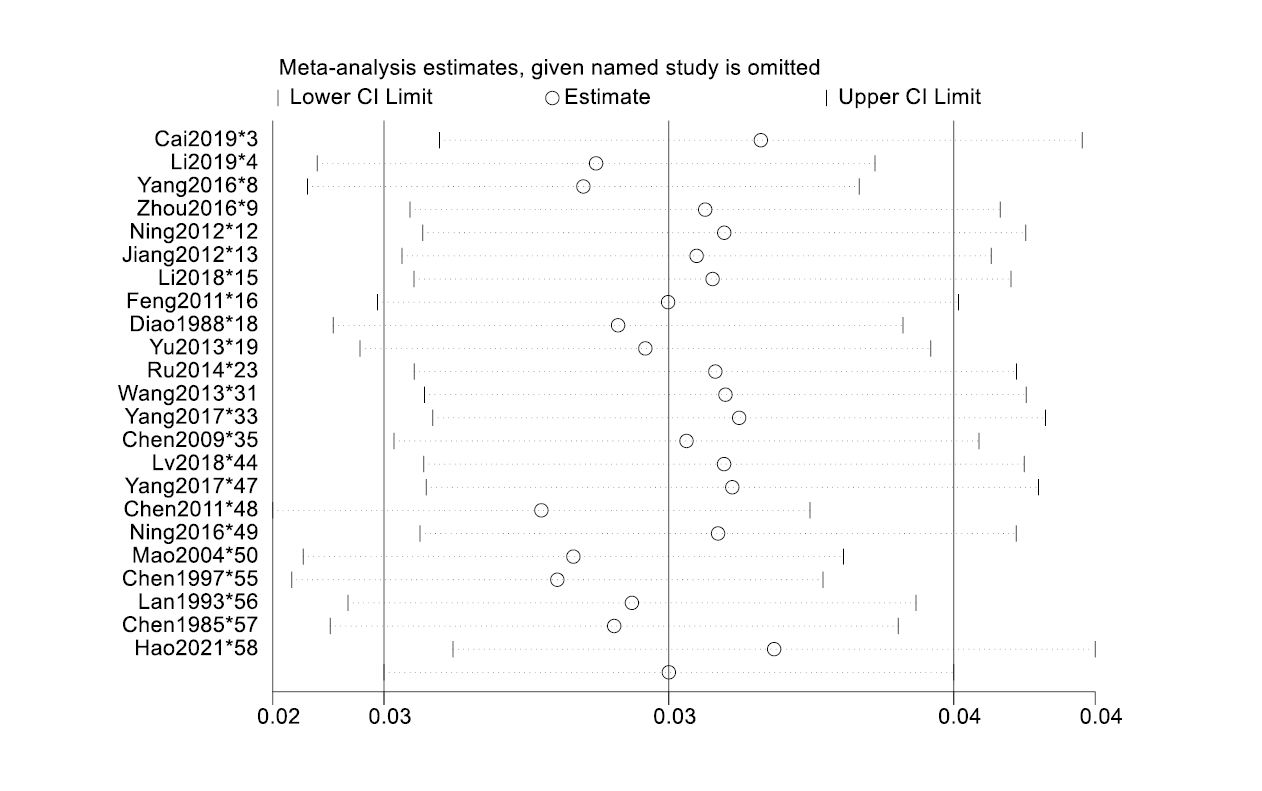


Supplementary Figure 3 Sensitivity analysis for in-hospital mortality of TSCI in China


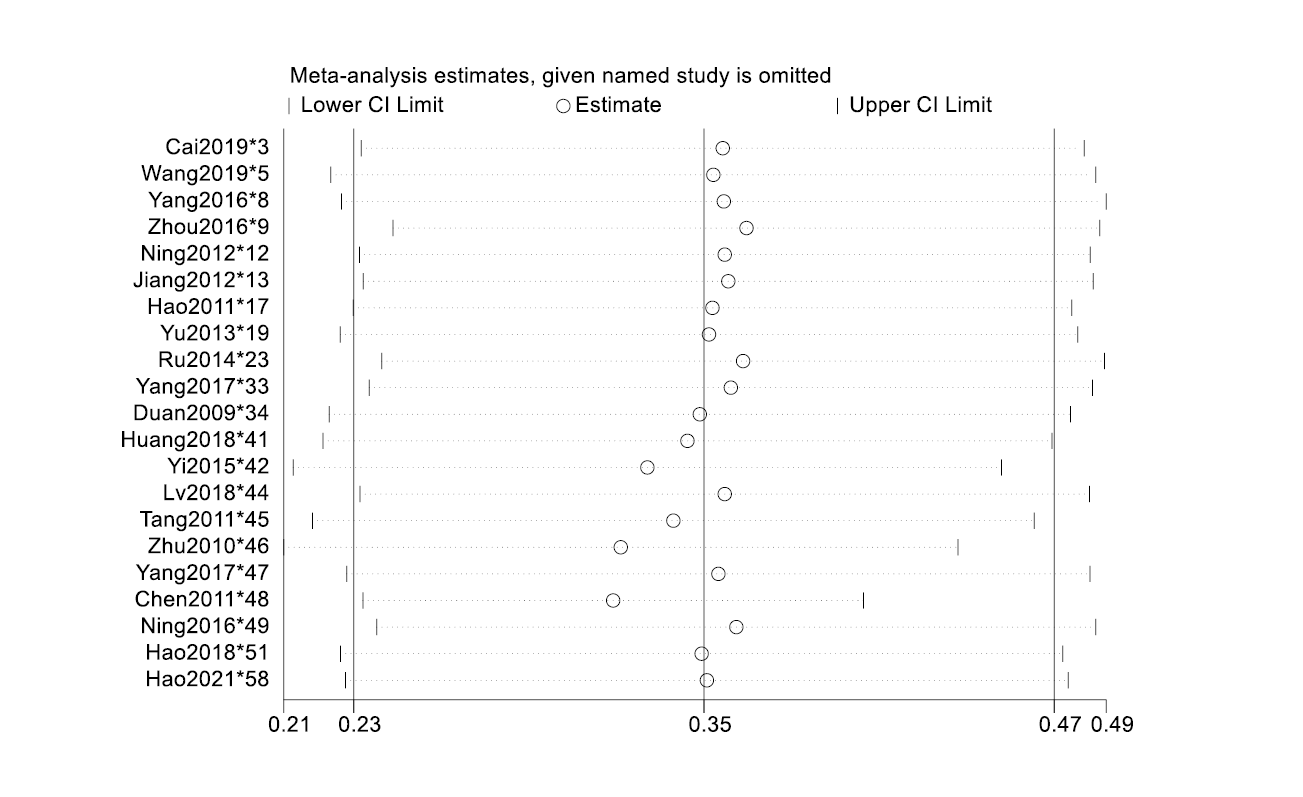


Supplementary Figure 4 Sensitivity analysis for complication rate of TSCI in China

Supplementary Table 1. Search strategy

Search date: October 1, 2022

| Database | Search strategy | Results |
| --- | --- | --- |
| EMBASE | ('spinal cord injury'/exp OR 'spinal cord injury' OR 'traumatic spinal cord injury'/exp OR 'traumatic spinal cord injury') AND ('epidemiology'/exp OR epidemiology OR 'incidence'/exp OR incidence OR 'etiology'/exp OR etiology OR 'prevalence'/exp OR prevalence) AND ('china'/exp OR china) AND [humans]/lim | 1331 |
| PubMed | (("spinal cord injury" or "Traumatic spinal cord injury") and (epidemiology or incidence or etiology or prevalence) and China)) Filters: Humans | 924 |
| Web of Science | (("spinal cord injury" or "Traumatic spinal cord injury") and (epidemiology or incidence or etiology or prevalence) and China)) | 419 |
| EBSCO： | (("spinal cord injury" or "Traumatic spinal cord injury") and (epidemiology or incidence or etiology or prevalence) and China)) | 124 |
| CNKI | SU='脊髓损伤' AND SU='流行病学' | 636 |
| VIP | U='脊髓损伤' AND U='流行病学' | 99 |
| Wan Fang Data | U='脊髓损伤' AND U='流行病学' | 292 |

Supplementary Table 2. The AHRQ Tool for assessing the quality of cross-sectional studies

The AHRQ tool assessed the risk of bias from five domains: selection bias, implementation bias, follow-up bias, measurement bias, and reporting bias. And it consists of 11 items, with 1 point for "yes" and 0 points for "no" or "unclear". 0-3 points for poor quality, 4-7 for medium quality, and 8-11 for good quality.

| Components | Yes | No | Unclear |
| --- | --- | --- | --- |
| 1. Define the source of information (survey, record review) |  |  |  |
| 2.List inclusion and exclusion criteria for exposed and unexposed subjects (cases and controls) or refer to previous publications |  |  |  |
| 3. Indicate time period used for identifying patients |  |  |  |
| 4.Indicate whether or no subjects were consecutive if not population-based |  |  |  |
| 5.Indicate if evaluators of subjective components of study were masked to other aspects of the status of the participants |  |  |  |
| 6.Describe any assessments undertaken for quality assurance purposes (e.g., test/retest of primary out come measurements) |  |  |  |
| 7. Explain any patient exclusions from analysis |  |  |  |
| 8. Describe how confounding was assessed and/or controlled |  |  |  |
| 9.If applicable, explain how missing data were handled in the analysis |  |  |  |
| 10.Summarize patient response rates and completeness of data collection |  |  |  |
| 11. Clarify what follow-up, if any, was expected and the percentage of patients for which incomplete data or follow-up was obtained |  |  |  |

Supplementary Table 3 Results of the quality assessments

| Study*ref | 1 | 2 | 3 | 4 | 5 | 6 | 7 | 8 | 9 | 10 | 11 | Score |
| --- | --- | --- | --- | --- | --- | --- | --- | --- | --- | --- | --- | --- |
| Liu *1 | Y | Y | Y | Y | N | N | Y | Y | Y | N | N | 7 |
| Liu *2 | Y | Y | Y | Y | N | Y | N | Y | N | N | N | 6 |
| Cai *3 | Y | N | Y | Y | N | Y | N | N | N | U | N | 4 |
| Li *4 | Y | N | Y | N | N | N | Y | N | N | N | N | 3 |
| Wang *5 | Y | Y | Y | U | N | N | N | N | N | N | N | 3 |
| Liu *6 | Y | Y | Y | Y | N | Y | N | N | N | N | N | 5 |
| Yuan *7 | Y | N | Y | U | N | N | N | N | N | N | N | 2 |
| Yang *8 | Y | N | Y | Y | N | N | N | N | N | N | N | 3 |
| Zhou*9 | Y | Y | Y | Y | N | Y | N | Y | N | N | N | 6 |
| Xu *10 | Y | N | Y | U | Y | Y | N | N | N | N | N | 4 |
| Wang *11 | Y | N | N | N | N | N | N | Y | N | N | N | 2 |
| Ning*12 | Y | Y | Y | N | N | Y | Y | Y | Y | N | N | 7 |
| Jiang *13 | Y | N | Y | Y | N | Y | N | N | N | N | N | 4 |
| Hua *14 | Y | N | Y | Y | N | N | N | Y | N | N | N | 4 |
| Li *15 | Y | Y | Y | Y | N | N | Y | N | N | Y | N | 6 |
| Feng *16 | Y | N | Y | N | N | Y | Y | Y | N | N | N | 5 |
| Hao *17 | Y | N | Y | N | N | N | N | N | N | N | N | 2 |
| Diao *18 | Y | N | Y | Y | N | N | N | N | N | Y | Y | 5 |
| Yu *19 | Y | Y | Y | Y | N | N | N | N | N | N | N | 4 |
| Wei*20 | Y | N | Y | N | N | N | N | N | N | N | N | 2 |
| Li*21 | Y | N | Y | N | N | Y | N | N | N | N | N | 3 |
| Liu*22 | Y | Y | Y | Y | N | N | N | Y | N | N | N | 5 |
| Ru*23 | Y | N | Y | Y | N | Y | Y | Y | Y | N | N | 7 |
| Xu*24 | Y | N | Y | Y | Y | N | N | N | N | N | N | 4 |
| Chen*25 | Y | Y | Y | Y | N | Y | N | Y | N | N | N | 6 |
| Niu*26 | Y | N | Y | N | N | N | N | N | N | N | N | 2 |
| Tang*27 | Y | Y | Y | N | N | Y | N | Y | N | N | N | 5 |
| Feng*28 | Y | Y | Y | Y | N | N | N | Y | N | N | N | 5 |
| Wu*29 | Y | Y | Y | Y | N | Y | N | Y | N | N | N | 6 |
| Niu*30 | Y | N | Y | N | N | N | N | Y | Y | N | N | 4 |
| Wang*31 | Y | N | Y | Y | N | Y | N | Y | N | N | N | 5 |
| Pan*32 | Y | N | Y | N | N | N | N | N | N | N | N | 2 |
| Yang*33 | Y | Y | N | N | N | N | N | N | N | N | N | 2 |
| Duan*34 | Y | N | Y | U | N | N | N | N | N | N | N | 2 |
| Chen*35 | Y | N | Y | N | N | N | N | N | N | N | N | 2 |
| Hu*36 | Y | N | Y | U | N | N | N | N | N | N | N | 2 |
| Chen*37 | Y | N | Y | Y | N | Y | N | N | Y | N | N | 5 |
| Shun*38 | Y | Y | Y | U | N | N | N | N | N | N | N | 3 |
| Feng*39 | Y | N | Y | N | N | N | N | N | N | N | N | 2 |
| Zhang*40 | Y | Y | Y | N | N | N | N | N | N | N | N | 3 |
| Huang*41 | Y | N | Y | Y | N | Y | N | N | N | N | N | 4 |
| Yi*42 | Y | Y | Y | N | N | Y | N | Y | N | N | N | 5 |
| Deng*43 | Y | N | Y | Y | Y | Y | N | N | N | N | N | 5 |
| Lv*44 | Y | N | Y | Y | N | N | N | N | N | N | N | 3 |
| Tang*45 | Y | Y | Y | N | N | Y | N | Y | N | N | N | 5 |
| Zhu*46 | Y | N | Y | Y | Y | N | N | N | N | N | N | 4 |
| Yang*47 | Y | Y | Y | N | N | Y | Y | Y | N | N | N | 6 |
| Chen*48 | Y | N | N | N | N | Y | N | N | N | N | N | 2 |
| Ning*49 | Y | Y | Y | N | N | N | N | N | N | N | N | 3 |
| Mao*50 | Y | N | Y | N | N | N | N | N | N | N | N | 2 |
| Hao*51 | Y | Y | Y | N | N | Y | N | Y | N | N | N | 5 |
| Zhang*52A | Y | Y | Y | U | N | Y | N | Y | N | N | N | 5 |
| Yang*53 | Y | N | Y | N | N | Y | N | Y | N | N | N | 4 |
| Wu*54 | Y | N | Y | Y | N | N | Y | N | N | Y | Y | 6 |
| Chen*55 | Y | N | Y | N | N | N | N | N | N | N | N | 2 |
| Lan*56 | Y | N | Y | N | N | N | Y | N | N | N | N | 3 |
| Chen*57 | Y | N | Y | N | N | N | N | N | N | N | N | 2 |
| Zhang*52B | Y | Y | Y | Y | N | Y | N | Y | N | N | Y | 7 |
| Hao*58 | Y | Y | Y | N | N | Y | N | Y | N | Y | N | 6 |
| Jiang*59 | Y | N | Y | N | Y | Y | Y | N | N | N | Y | 6 |

Note: Y=yes, N=no, U=unclear

**References**

1. Liu J, Gao F, Li JJ. Epidemiology of patients with traumatic spinal cord injury and study on the influencing factors of hospitalization costs. [Chinese]. Chinese Journal of Rehabilitation. 2020,35(03):139-142.
2. Liu HW, Liu J, Shen MX, Yang XH, Du LJ, Yang ML, et al. The changing demographics of traumatic spinal cord injury in Beijing, China: a single-centre report of 2448 cases over 7 years. Spinal Cord. 2021;59(3):298-305.
3. Cai ZW, Regional Epidemiological Investigation of Traumatic Spinal Cord Injury. [Chinese] [Master]: Tianjin Medical University; 2019.
4. Li WX, Li RF, Yu Bl. Epidemiological analysis of 956 inpatients with traumatic spinal cord injury from 2012 to 2019. [Chinese]. Chinese Journal of Spine and Spinal Cord. 2021,31(07):626-631.
5. Wang L, Zhou J, Shi XX, Hu Y, Qin J, Yin JK, et al. Advances in studies on the factors related to traumatic spinal cord injury. [Chinese]. Chinese Journal of Bone and Joint. 2017,6(02):139-144.
6. Liu J, Liu HW, Gao F, Li J, Li JJ. Epidemiological features of traumatic spinal cord injury in Beijing, China. J Spinal Cord Med. 2022;45(2):214-220.
7. Yuan YK. Epidemiological analysis of patients with spinal cord injury. [Chinese]. Grassroots Medical Forum. 2015,19(30):4316-4317.
8. Yang XX, Yu QJ, Qin J, Li ZH, Song KR, Ren DF, et al. Epidemiological analysis of 1027 inpatients with spinal cord injury. [Chinese]. J Spinal Surg. 2016,14(05):301-305.
9. Zhou Y, Wang XB, Kan SL, Ning GZ, Li YL, Yang B, et al. Traumatic spinal cord injury in Tianjin, China: a single-center report of 354 cases. Spinal Cord. 2016;54(9):670-674.
10. Xu Q, Yuan L, Gao F, Zhou HJ, Liu WG, LI JJ, et al. Investigation and analysis of anorectal and perianal diseases in patients with spinal cord injury. [Chinese]. Proceedings of the 14th Congress of Chinese Association of Spinal Cord. 2012:63-65.
11. Wang PS, Wang S, Liu XB, An YH. Analysis of the causes and clinical characteristics of traumalicspinal cord injury based on 1395 cases. [Chinese]. Chin J Emerg Resuse Disaster Med. 2020,15(3):340-344.
12. Ning GZ. Study on Epidemiology of Spinal Cord Injury in Tianjin. [Chinese] [Doctor]: Tianjin Medical University; 2012.
13. Jiang JC, Zhu LQ, Ye CQ, Sun TS, Xu ST. Characteristics of Spinal Cord Injury in Hospital: 423 Cases Report. [Chinese]. Chin J Rehabil Theory Pract. 2012,18(07):665-668.
14. Hua R, Shi J, Wang X, Yang J, Zheng P, Cheng H, et al. Analysis of the causes and types of traumatic spinal cord injury based on 561 cases in China from 2001 to 2010. Spinal Cord. 2013;51(3):218-221.
15. Li HL. A Single Center Study on Epidemiological Characteristics of Spinal Cord Injury during 1999-2016 in Tianjin. [Chinese] [Master]: Tianjin Medical University; 2018.
16. Feng HY, Ning GZ, Feng SQ, Yu TQ, Zhou HX. Epidemiological profile of 239 traumatic spinal cord injury cases over a period of 12 years in Tianjin, China. J Spinal Cord Med. 2011;34(4):388-394.
17. Hao CX, Li JJ, Zhou HJ, Kang HQ, Li SQ, Liu GL, et al. Epidemiology Characteristics of Spinal Cord Injury in Hospital: 1264 Cases Report. [Chinese]. Chinese Journal of Rehabilitation Theory and Practice. 2007(11):1011-1013.
18. Beijing Spinal Cord Injury Investigation Group. A five-year retrospective survey of spinal cord injury in beijing. [Chinese]. Chinese Journal of Rehabilitation.1988(02):59-62.
19. Yu TQ.Epidemiology of Traumatic Spinal Cord Injuries in Urban of Tianjin in 2007. [Chinese] [Master]: Tianjin Medical University; 2010.
20. Wei B. The Primary Investigation into the Epidemiology of Spinal Cord Injury in Beijing in 2005. [Chinese] [Doctor]: Capital Medical University; 2007.
21. Li JJ, Zhou HJ, Hong Y, Ji JP, Liu GL, Li SQ, et al. Spinal cord injuries in Beijing: a municipal epidemiological survey in 2002. [Chinese]. Chinese Journal of Rehabilitation Theory and Practice. 2004(07):32-33.
22. Liu L. Reginal Retrospective Investigation of Spinal Cord Injury Epidemiology. [Chinese] [Master]: Tianjin Medical University; 2019.
23. Ru QC. Epidemiological Study of Spinal Cord Injury in Dalian. [Chinese] [Master]: Dalian Medical University; 2014.
24. Xu CG, Gu R, Wang TB, Jiang BG. Epidemiological analysis of 1274 cases of spinal trauma. [Chinese]. JIN RI JIAN KANG. 2016,15(11):19-20.
25. Chen R, Liu X, Han S, Dong D, Wang Y, Zhang H, et al. Current epidemiological profile and features of traumatic spinal cord injury in Heilongjiang province, Northeast China: implications for monitoring and control. Spinal Cord. 2017;55(4):399-404.
26. Niu SJ, Zhou QQ, Zhang DW. Epidemiological analysis of 422 hospitalized patients with traumatic spinal cord injury. [Chinese]. Bao Jian Wen Hui. 2021,22(31):21-23.
27. Tang YL. Study of the status of urinary tract infection and bladder management and related risk factors in patients with spinal cord injury. [Chinese] [Master]: Qingdao University; 2021.
28. Feng H, Xu H, Zhang H, Ji C, Luo D, Hao Z, et al. Epidemiological profile of 338 traumatic spinal cord injury cases in Shandong province, China. Spinal Cord. 2022;60(7):635-640.
29. Wu F, Zheng Y, Ren B, Huang L, Yang D. Current epidemiological profile and characteristics of traumatic cervical spinal cord injury in Nanchang, China. J Spinal Cord Med. 2022;45(4):556-563.
30. Niu SJ. Epidemiologic analysis of spinal cord injury in Suzhou and clinical study of related factors. [Chinese] [Master]: Soochow University; 2016.
31. Wang HF, Yin ZS, Chen Y, Duan ZH, Hou S, He J. Epidemiological features of traumatic spinal cord injury in Anhui Province, China. [Chinese]. Spinal Cord. 2013;51(1):20-2.
32. Pan J, Li X, Zeng C, Qian L, Li LJ, Tan J. Retrospective study of acute spinal cord injury between 2005 and 2007 in Pudong New Area, Shanghai. [Chinese]. Journal of Tongji University(Medical Science). 2009,30(05):131-135.
33. Yang WQ. Epidemiological Investigation Of 1089 Hospitalized Patients With Spinal Cord Injury. [Chinese] [Master]: Fujian Medical University; 2015.
34. Duan MS, Shu Y, Cao K, Han ZM, Huang SH. Clinical analysis of early complications and related factors in 650 patients with acute spinal cord injury. [Chinese]. Chinese Journal of Physical Medicine and Rehabilitation. 2009(09):632-634.
35. Chen J, Chen BH. Clinical analysis of 251 cases of traumatic spinal cord injury. [Chinese]. Chinese Community Doctors. 2009,11(19):69.
36. Hu GY, Tang HF, Tang LA. Epidemiological investigation of spinal cord injury in Songjiang County, Shanghai. [Chinese]. Chin J Spine Spinal Cord. 1992(04):177-179.
37. Cheng LM, Zeng ZL, Yang ZY, Zhou JL, Yuan F, Zhang SM, et al. Epidemiologic features and effects of surgical treatment of spinal injudes treated in one medicaI center. [Chinese]. Chin J Orthop Trauma. 2008(03):245-248.
38. Sun ZY, Chen BH, Hu YG, Ma XX, Yuan B. The Value of China National Spinal Cord Injury Database in the Analysis of Patients with Acute Apinal Cord Injury. [Chinese]. Acta Aacademiae Medicinae Qingdao Universitatis. 2012,48(02):115-117.
39. Pang QN. Epidemiological survey of traumatic SCI in Wuxi in 1991. [Chinese]. Chin J Spine Spinal Cord. 1993, (6):266.
40. Zhang SP, Cao LJ, Zhou BQ, Zhong C. Analysis of the characteristics of acute spinal cord injury in the urbanrural fringe of Guangzhou. [Chinese]. China Modern Medicine. 2020,27(29):163-165+184.
41. Huang Y, Ye L, Fen HY, Liu WW. Epidemiology Characteristics of 397 cases of Spinal Cord Injury in Hospital. [Chinese]. Chinese Manipulation & Rehabilitation Medicine. 2018,9(20):38-40.
42. Yi CR. Epidemiological Features of 261 Hospitalized Patients with Spinal Cord Injury from Multiple Hospitals in Hunan Province. [Chinese] [Master]: University of South China; 2015.
43. Deng L, Shang H, Chang W, Wu YP, Li BK, Guo ZK, et al. Epidemiologic analysis of 424 cases of spine and spinal cord injuries. [Chinese]. Chinese Journal of Clinical Research. 2015,28(07):858-860+864.
44. Lv DB. Epidemiological Investigation of Spinal Cord Injury. [Chinese] [Master]: Zhengzhou University; 2018.
45. Tang ZS. Epidemiological Characteristics and Correlative Analysis of Inpatients with Spinal Cord Injury :221 Cases Report. [Chinese] [Master]: Guangxi Medical University; 2011.
46. Zhu CJ. Clinical Observation on Early Rehabilitation Treatment of Spinal Cord Injury. [Chinese] [Master]: Central South University; 2010.
47. Yang R, Guo L, Huang L, Wang P, Tang Y, Ye J, et al. Epidemiological Characteristics of Traumatic Spinal Cord Injury in Guangdong, China. Spine (Phila Pa 1976). 2017;42(9):E555-E561.
48. Chen YH, Liu M, He JH. Epidemiological survey of patients with spinal cord injury. [Chinese]. Chinese Journals of Practical Medicine. 2011,27(06):1032-1034.
49. Ning GZ, Mu ZP, Shangguan L, Tang Y, Li CQ, Zhang ZF, et al. Epidemiological features of traumatic spinal cord injury in Chongqing, China. J Spinal Cord Med. 2016;39(4):455-60.
50. Mao Q, Liu YH, Mao BY. Analysis of the Associated Factors of Spinal Cord Injuries with Multi┐traumas. [Chinese]. Journal of Sichuan University(Medical Sciences). 2004(02):244-246.
51. Hao DJ, He BR, Yan L, Wang Y, Zhang Q, Liu CC, et al. Epidemiological profile of spinal cord injury from 2011 through 2013 at Xi’an Honghui Hospital. [Chinese]. Chinese Journal of Traumatology. 2015,31(07):632-636.
52. Zhang JJ. The Disease Burden and Outcome of Hospitalized Patients with Traumatic Spinal Cord Injury in China. [Chinese] [Master]: Chinese Center for Disease Control and Prevention; 2021.
53. Yang NP, Deng CY, Lee YH, Lin CH, Kao CH, Chou P. The incidence and characterisation of hospitalised acute spinal trauma in Taiwan--a population-based study. Injury. 2008;39(4):443-50.
54. Wu JC, Chen YC, Liu L, Chen TJ, Huang WC, Cheng H, et al. Effects of age, gender, and socio-economic status on the incidence of spinal cord injury: an assessment using the eleven-year comprehensive nationwide database of Taiwan. J Neurotrauma. 2012;29(5):889-97.
55. Chen HY, Chiu WT, Chen SS, Lee LS, Hung CI, Hung CL, et al. A nationwide epidemiological study of spinal cord injuries in Taiwan from July 1992 to June 1996. Neurol Res. 1997;19(6):617-22.
56. Lan C, Lai JS, Chang KH, Jean YC, Lien IN. Traumatic spinal cord injuries in the rural region of Taiwan: an epidemiological study in Hualien county, 1986-1990. Paraplegia. 1993;31(6):398-403.
57. Chen CF, Lien IN. Spinal cord injuries in Taipei, Taiwan, 1978-1981. Paraplegia. 1985;23(6):364-70.
58. Hao D, Du J, Yan L, He B, Qi X, Yu S, et al. Trends of epidemiological characteristics of traumatic spinal cord injury in China, 2009-2018. Eur Spine J. 2021;30(10):3115-3127.
59. Jiang B, Sun D, Sun H, Ru X, Liu H, Ge S, et al. Prevalence, Incidence, and External Causes of Traumatic Spinal Cord Injury in China: A Nationally Representative Cross-Sectional Survey. Front Neurol. 2022;12:784647.
